# Subglottic secretion suction for preventing ventilator-associated pneumonia: an updated meta-analysis and trial sequential analysis

**DOI:** 10.1186/s13054-016-1527-7

**Published:** 2016-10-28

**Authors:** Zhi Mao, Ling Gao, Guoqi Wang, Chao Liu, Yan Zhao, Wanjie Gu, Hongjun Kang, Feihu Zhou

**Affiliations:** 1 Department of Critical Care Medicine, Chinese People’s Liberation Army General Hospital, 28 Fu-Xing Road, Beijing, 100853 People’s Republic of China; 2 Department of Cardiovascular Surgery, Institute of Cardiac Surgery, Chinese People’s Liberation Army General Hospital, Beijing, People’s Republic of China; 3 Department of Orthopaedics Chinese, People’s Liberation Army General Hospital, Beijing, People’s Republic of China; 4 Department of Anesthesiology, Drum Tower Hospital, Medical College of Nanjing University, Nanjing, China

**Keywords:** Subglottic secretion suction, Ventilator-associated pneumonia, Meta-analysis, Trial sequential analysis

## Abstract

**Background:**

Potential benefits of subglottic secretion suction for preventing ventilator-associated pneumonia (VAP) are not fully understood.

**Methods:**

We searched Cochrane Central, PubMed, and EMBASE up to March 2016 to identify randomized controlled trials (RCTs) that compared subglottic secretion suction versus non-subglottic secretion suction in adults with mechanical ventilation. Meta-analysis was conducted using Revman 5.3, trial sequential analysis (TSA) 0.9 and STATA 12.0. The primary outcome was incidence of VAP. The Grading of Recommendations Assessment, Development, and Evaluation (GRADE) was used to evaluate the level of evidence.

**Results:**

Twenty RCTs (*N* = 3544) were identified. Subglottic secretion suction was associated with reduction of VAP incidence in four high quality trials (relative risk (RR) 0.54, 95 % confidence interval (CI) 0.40–0.74; *p* < 0.00001) and in all trials (RR = 0.55, 95 % CI 0.48– 0.63; *p* < 0.00001). Sensitivity analyses did not show differences in the pooled results. Additionally, the results of the above-mentioned analyses were confirmed in TSA. GRADE level was high. Subglottic secretion suction significantly reduced incidence of early onset VAP, gram-positive or gram-negative bacteria causing VAP, and duration of mechanical ventilation. It delayed the time-to-onset of VAP. However, no significant differences in late onset VAP, intensive care unit (ICU) mortality, hospital mortality, or ICU length of stay were found.

**Conclusions:**

Subglottic secretion suction decreased VAP incidence and duration of mechanical ventilation and delayed VAP onset. However, subglottic secretion suction did not reduce mortality and length of ICU stay. Subglottic secretion suction is recommended for preventing VAP and for reducing ventilation length, especially in the population at high risk of early onset VAP.

**Trial registration:**

A protocol of this meta-analysis has been registered on PROSPERO (registration number: CRD42015015715); registered on 5 January 2015.

**Electronic supplementary material:**

The online version of this article (doi:10.1186/s13054-016-1527-7) contains supplementary material, which is available to authorized users.

## Background

Ventilator-associated pneumonia (VAP) is a common clinical issue for patients receiving mechanical ventilation in the intensive care unit (ICU) [1]. The incidence of VAP accounts for 9 % to 27 % endotracheal intubated patients, whereas VAP has a mortality rate ranging from 25 % to 50 % [2–4]. VAP increased ICU and hospital length of stay, antibiotic consumption, and healthcare cost [5–8]. The primary mechanism of VAP is the microaspiration of the accumulated secretions around the endotracheal tube cuff. Preventive measures for microaspiration and VAP include continuous control of tracheal cuff pressure, conical cuff shape, and subglottic secretion suctioning (SSS).

SSS has been recommended in several guidelines to prevent the occurrence of VAP [9, 10]. Previous meta-analyses reported that SSS was associated with a lower rate of VAP [11–16]. However, many ICUs did not use SSS as a part of the VAP bundle [17]. Although about 55 % of hospitals in the US routinely used SSS in 2013 [18], European consensus did not recommend SSS for VAP prevention [19]. The prevention of VAP by SSS is not fully proven based on current evidence. The effect of SSS on early- or late-onset VAP, duration of mechanical ventilation, ICU length of stay, and mortality is controversial [16]. The need for future randomized controlled trials (RCTs) is still advocated [15, 16]. Six RCTs [17, 20–24] involving new evidence on this topic have been published recently.

Therefore, we conducted an updated meta-analysis of RCTs to evaluate the effect of SSS on VAP prevention. Trial sequential analysis (TSA) was used to determine whether the currently evidence was robust and conclusive.

## Methods

The Preferred Reporting Items for Systematic Reviews and Meta-Analyses (PRISMA) were used to report this systematic review and meta-analysis [25]. A protocol for this meta-analysis has been registered on PROSPERO (http://www.crd.york.ac.uk/prospero), and the registration number is CRD42015015715.

### Eligibility criteria

The inclusion criteria were as follows: 1) patients received mechanical ventilator (≥48 h); 2) intervention was SSS regardless of continuous or intermittent SSS; 3) the control was the non-subglottic secretion suctioning group; 4) the incidence of VAP was reported; and 5) the study design was RCT. The exclusion criteria were patients younger than 18 years and repeated data.

### Search strategy

A literature search of Cochrane Central (March 2016), PubMed (1950 to March 2016), and EMBASE (1980 to March 2016) was undertaken to identify trials according to Cochrane Collaboration methodology. The search terms used were “ventilator-associated pneumonia” with “subglottic secretion” or “subglottic drainage” or “subglottic suctioning” or “glottic” and “randomized” or “randomised”. No language restriction was applied. We also hand-searched the reference lists of review articles and conference proceedings.

### Study selection

Two independent reviewers (ZM and LG) conducted the study selection. Disagreements between the two reviewers were resolved in meetings or adjudicated by a third reviewer (GW).

### Data extraction

For each included study, data extraction was performed independently by two reviewers using a standard form. The following data on study characteristics were collected: year of publication, the study type, number of patients, patient characteristics, method of SSS, definition of VAP, incidence of VAP, length of ICU stay, duration of mechanical ventilation (MV), mortality, airway secretion bacteria detection, and details of the outcomes. The quality of included studies was assessed by the Cochrane risk of bias tool [26]. Trials with low risk of bias in all items were evaluated as high-quality studies.

### Grading the quality of evidence

Grading of Recommendations Assessment, Development, and Evaluation (GRADE) methodology was used to assess the quality of evidence classified as high, moderate, low, or very low. The judgments were based on risk of bias, inconsistency, indirectness, imprecision, and publication bias. GRADE Pro-version 3.6 software was used.

### Statistical analysis

Dichotomous variables were presented as relative risks (RRs), and continuous variables were presented as mean difference (MD), both with 95 % confidence intervals (CIs). Heterogeneity across studies was evaluated by visual inspection of the forest plot, along with quantification using the *I* ^2^ statistic. An *I* ^2^ > 50 % was considered as significant heterogeneity. A fixed-effects model was used in the meta-analysis unless significant heterogeneity among studies was present. The random-effects model of DerSimonian and Laird [27] was used regardless of heterogeneity. Statistical analysis was performed with Review Manager 5.1 software (Cochrane Collaboration, Oxford, UK) for outcome measures. A *p* value <0.05 was considered statistically significant. We assessed publication bias by visually inspecting a funnel plot. The Begg and Egger tests were also conducted using STATA 12.0.

For the primary result, predefined subgroup analyses were performed as the high-quality subgroup, and the primary result from high-quality trials was emphasized. Sensitivity analysis for primary results were conducted by excluding trials with multiple manipulations, continuous versus intermittent suction, appropriate randomization, allocation concealment, assessment blinding, and participants numbering more than 100. We also performed sensitivity analyses using an invasive diagnosis of VAP in all outcomes.

The second outcomes were incidence of early- or late-onset VAP, gram-positive or gram-negative bacteria causing VAP, ICU or hospital mortality, time-to-onset of VAP, duration of mechanical ventilation, ICU or hospital length of stays, and incidence of tracheotomy or reintubation.

### Trial sequential analysis

TSA, which is similar to an interim trial analysis in a single trial, was conducted to obtain the primary result. Cumulative meta-analysis that is updated with new studies may result in false positive results (type I error) because of an increased risk of random error from sparse data and repeated significance testing [28]. TSA can control the *p* value and widen the confidence intervals [29]. TSA combined concepts and rationale as follows: an estimation of the required information size and trial sequential monitoring boundaries. If the cumulative Z curve enters the futility area or crosses the trial sequential monitoring boundary, the anticipated intervention effect may reach a sufficient level of evidence, and further trials will not be necessary. If the Z curve does not cross any of the boundaries or reach the required information size, evidence is insufficient for drawing a conclusion.

We calculated the required information size based on a relative risk reduction of 20 % in incidence of VAP. The type I error (α) and power (1 – β) were set as 0.05 and 0.80, respectively. The control event rates were calculated from the non-subglottic secretion suctioning group. The TSA was conducted with the use of TSA version 0.9 beta software (http://www.ctu.dk/tsa).

## Results

### Trial selection

**Fig. 1:**
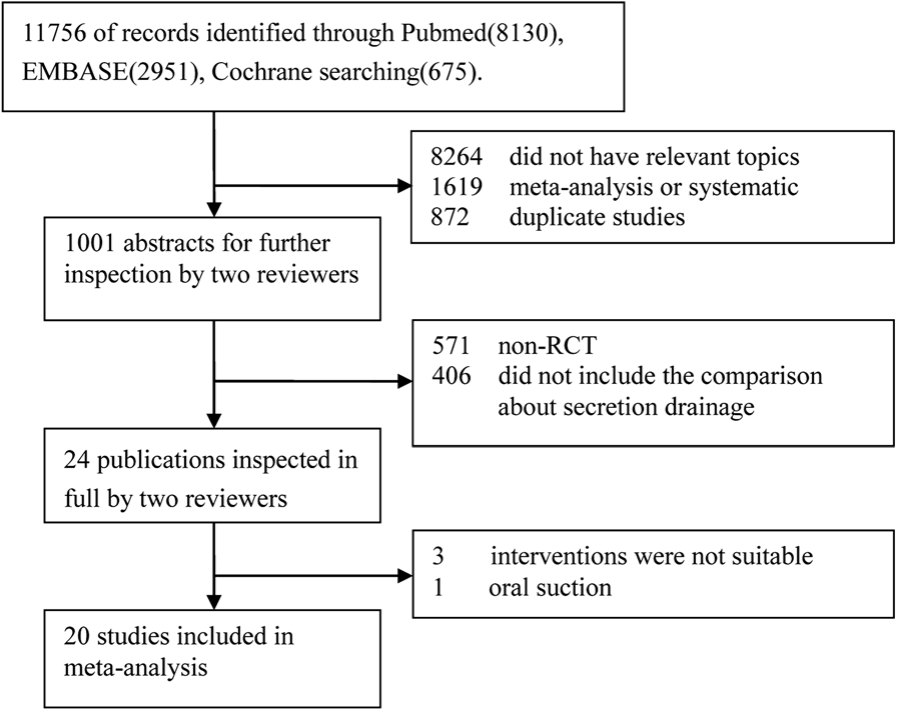
Flow chart of the trial selection. *RCT* randomized controlled trial

A total of 11,756 potentially relevant articles were used. We excluded duplicate studies, non-relevant topic articles, non-RCTs, and non-suitable intervention studies. Twenty studies reported that 3544 patients were included in this meta-analysis (Fig. 1) [17, 20–24, 30–43].

### Trials characteristics

The main characteristics of the selected studies are summarized in Table 1. These studies were reported between 1992 and 2016, and a total of 3544 patients were included. Fourteen studies were published in English [17, 20, 21, 23, 24, 30–32, 34–36, 38, 39, 42], five in Chinese [22, 33, 37, 40, 41], and one in Persian [43]. One abstract was included [24].

**Table 1:** Characteristics of included studies

Study	Population (*n*)	Settings	Definition of VAP	Inclusion criteria	Exclusion criteria	Method of SSS	Antibiotic use	Evaluation unit of antibiotic consumption
Mahul, 1992 [30]	145	Medical-surgical ICU	Positive bronchoalveolar lavage culture required	Expected duration of MV >3 days	Gastrointestinal bleeding, tracheostomy, risk of reintubation, intubated before ICU	Intermittent	Antibiotic use at randomization not reported	NA
Valles, 1995 [31]	153	Medical-surgical ICU	Clinical features confirmed with bronchoscopically obtained cultures	Expected duration of MV >3 days	Intubated before arriving at the emergency department or ICU; tracheostomy	Continuous	Patients receiving antibiotics at time of randomization: 64 % and 58 %, intervention and control group respectively	NA
Kollef, 1999 [32]	343	Cardiothoracic ICU	Clinical features, positive tracheal, blood, or pleural cultures; radiographic abscess, or positive histology	Need for MV after cardiac surgery	Intubated before ICU; transfer from outside hospital	Continuous	Antibiotics were given to 99 % and 98 % of intervention and control patients, respectively	NA
Bo, 2000 [33]	68	Surgical ICU	Clinical features or positive blood/pleural cultures	Expected duration of MV >72 h	Intubated outside hospital; high-risk surgery or trauma; pre-existing infection	Continuous	Antibiotics were used in 29 % of intervention group and 36 % of control group	NA
Smulders, 2002 [34]	150	Medical-surgical ICU	Clinical features or positive blood/pleural cultures	Expected duration of MV >72 h	Patients expected to require >72 h MV	Intermittent	48 % of intervention group and 51 % of control group were receiving antibiotics	NA
Girou, 2004 [35]	18	NA	Clinical features and significant quantitative culture of aspiration	Expected duration of MV >5 days	NA	Continuous	Prior antibiotic therapy: 1 patient in suctioning group and 4 patients in control group	NA
Liu SH, 2006 [37]	98	Respiratory ICU	MV >48 h, the chest X-ray showed pulmonary new or progressive infiltration lesions, and excluding atelectasis, pulmonary edema, and pleural effusion	Age older than 60 years, expected MV >48 h	NA	Intermittent	NA	NA
Liu QH, 2006 [36]	86	NA	Received MV for >48 h, clinical features and culture of endotracheal aspirate; reduction of oxygen	Age older than 60 years, expected MV >48 h	1) expected death within 48 h; 2) expected weaning within 48 h; 3) existing lung infection when MV beginning	Continuous	NA	NA
Lorente, 2007 [38]	280	NA	Clinical features and significant quantitative culture via ETT aspiration	Expected MV >24 h	Age less than 18 years, pregnancy, infection with human immunodeficiency virus, blood leukocyte count less than 1000 cells/mm3, solid or hematological tumor, and/or immunosuppressive therapy	Continuous	83.6 % of ETT-PUC-SSD group and 85 % of ETT-C group were receiving antibiotics after cardiothoracic surgery	NA
Bouza, 2008 [39]	704	Cardiothoracic ICU	Received MV for >48 h, clinical features and culture of endotracheal aspirate; reduction of oxygen	Major heart surgery	NA	Continuous	Both group respectively received antibiotics before surgery and every 8 h thereafter for a total of three doses in the ICU	Daily defined doses
Yang, 2008 [40]	91	Medical-surgical ICU	Clinical features and culture of endotracheal aspirate	MV >48 h	Intubated before ICU	Continuous	NA	NA
Zheng, 2008 [41]	61	Medical-surgical ICU	NA	MV >48 h	NA	Continuous	NA	NA
Lacherade, 2010 [42]	333	Multicenter, medical-surgical ICU	Quantitative culture of protected telescoping catheter sample or bronchoalveolar lavage fluid following clinical suspicion	Expected MV >48 h	Intubated before ICU; tracheostomy; psychotropic drug overdose; acute drunkenness; cardiac arrest	Intermittent	Antibiotics therapy was used in 94 % and 92 % of intervention and control groups respectively	NA
Seyfi, 2013 [43]	80	ICU of Hazrat Rasool Akram Hospital of Tehran, Iran.	NA	NA	NA	Intermittent	NA	NA
Safdari, 2014 [23]	76	In four ICUs of Educational Hospital in Isfahan, Iran	NA	Intubated with a conventional endotracheal tube and connected to ventilators for more than 48 h	Patients who were admitted to the ICUs with tracheostomy or likely to die in the next 48 h or admitted to these units for treatment of pneumonia or with lung complications like fibrosis or cancer	Intermittent performed every 3 h	NA	NA
Koker, 2014 [24]	51	In a 14-bed ICU	NA	Requiring prolonged MV for more than 48 h	NA	Continuous	NA	NA
Tao, 2014 [22]	149	NA	Received MV for >48 h, clinical features and culture of endotracheal aspirate; reduction of oxygen	Expected MV >48 h with APACHE score 20–30	Existing lung infection when MV beginning	Continuous or intermittent every 4 h	NA	NA
Damas, 2015 [17]	352	All ICUs of a tertiary hospital	Clinical features and culture of endotracheal aspirate	Age over 18 years, intubation with a Teleflex Isis™ endotracheal tube (TIET) allowing subglottic secretion suctioning	Patient participating in another study or having already participated in this study	NA	NA	The numberof antibiotic days
Gopal, 2015 [21]	240	Cardiothoracic surgery	Europe Infection Control through Surveillance definition	Age over 70 years and/or left ventricular ejection fraction <50 % and cardiac surgery	NA	Intermittent every 6 h	NA	NA
Deem, 2016 [20]	66	All ICUs at Harborview Medical Center	Criteria of Center for Disease Control	Age over 18 years with intubation and needed critical care	1) Out-of-hospital cardiac arrest; 2) non-study designated intubation devices; 3) airway management other than orotracheal intubation; 4) Federally protected populations, pregnant women, and prisoners	Continuous	NA	NA

*APACHE* Acute Physiology and Chronic Health Evaluation, *ETT-C* conventional endotracheal tube, *ETT-PUC-SSD* endotracheal tube with polyurethane cuff and subglottic secretion drainage, *ICU* intensive care unit, *MV* mechanical ventilation, *NA* not available, *SSS* subglottic secretion suctioning, *VAP* ventilator-associated pneumonia

### Risk of bias assessment

**Fig. 2:**
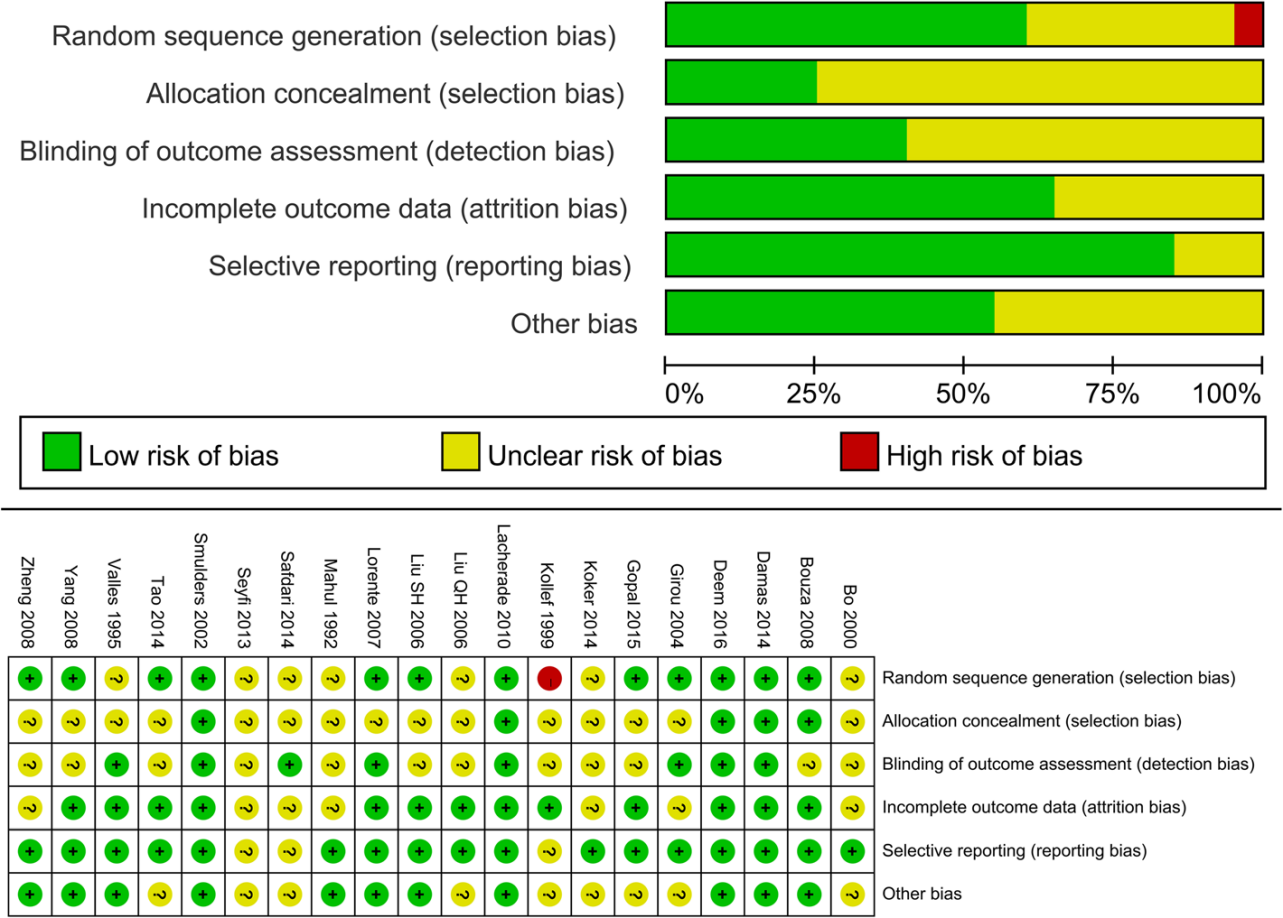
Risk of bias table

Risk of bias is summarized in Fig. 2. Twelve studies reported and adequate randomized sequence generated [17, 20–22, 34–36, 38–42], five studies reported appropriate allocation concealment [17, 20, 34, 39, 42], and seven studies reported blinding of outcome assessments [17, 23, 31, 34, 35, 38, 42]. Four studies were high-quality studies with low risk of bias in all items (Fig. 2) [17, 20, 34, 42].

### Primary outcome: incidence of VAP

**Fig. 3:**
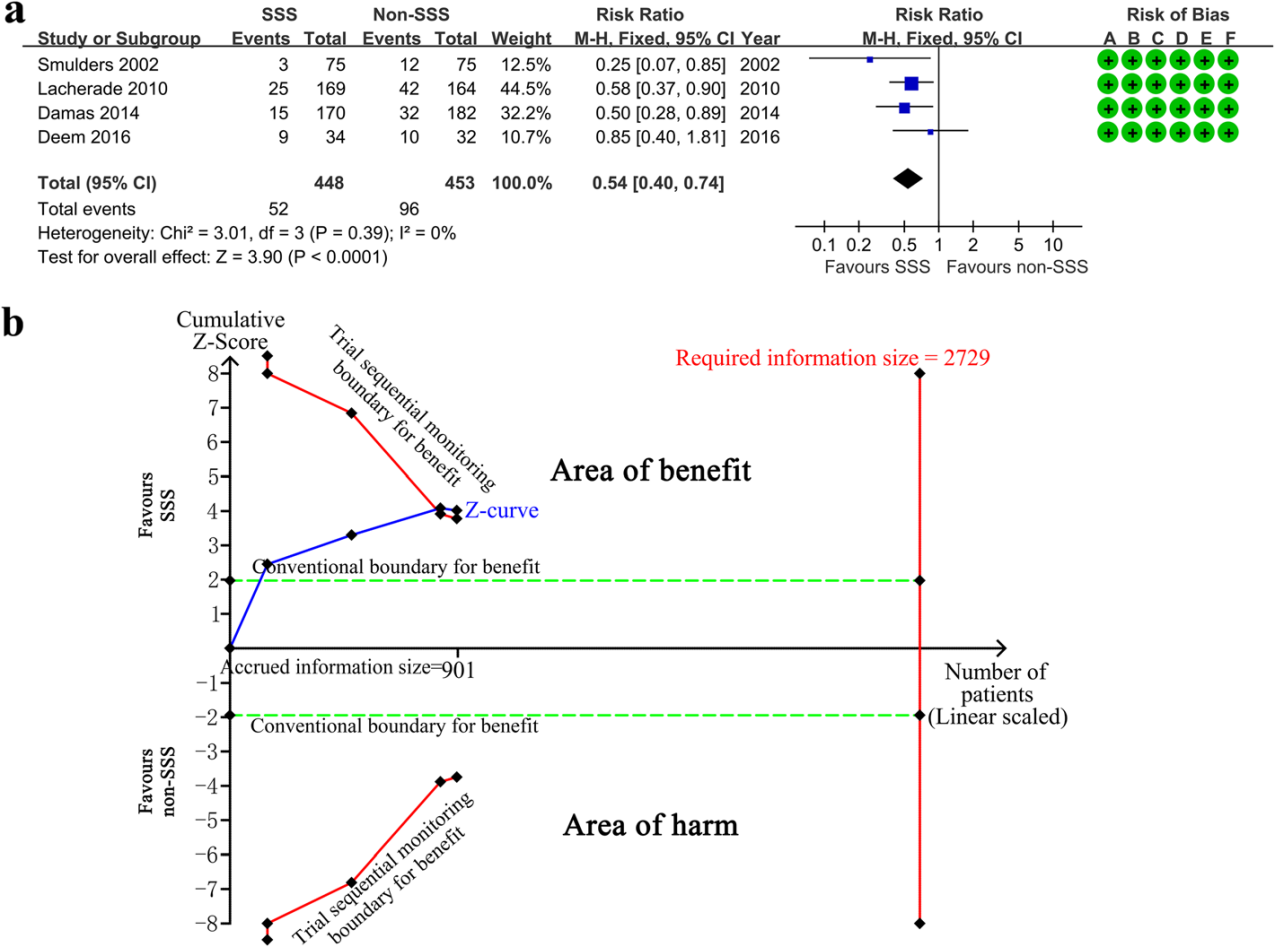
Effect of subglottic secretion suction on preventing ventilator-associated pneumonia in four high-quality trials. **a** Forest plot comparing subglottic secretion suction (*SSS*) and non-SSS on incidence of ventilator-associated pneumonia. **b** Trial sequential analysis for incidence of ventilator-associated pneumonia with control arm event proportion of 21.2 %, relative risk reduction of 20 %, α of 5 % (two sided), β of 20 %, and *I* ^2^ = 0 %. The required information size was calculated as 2729. Z curve has across-trial sequential monitoring boundary for benefit. Risk of bias: *A* random sequence generation (selection bias), *B* allocation concealment (selection bias), *C* blinding of outcome assessment (detection bias), *D* incomplete outcome data (attrition bias), *E* selective reporting (reporting bias), *F* other bias; *CI* confidence interval, *M-H* Mantel-Haenszel

Four high-quality studies with 901 participants were included in the analysis of VAP incidence (Fig. 3) [17, 20, 34, 42], thereby suggesting an RR of 0.54 (95 % CI 0.40–0.74; *p* < 0.00001; *p* for heterogeneity = 0.39, *I* ^2^ = 0 %) for SSS versus non-SSS. Absolute risk reduction (ARR) was 0.0953. The number needed to treat (NNT) was 10.49. TSA showed that the cumulative Z curves crossed both the conventional boundary and the trial sequential monitoring boundary. Thus, further trials were unlikely to change the conclusion (Fig. 3, Table 2).

**Table 2:** The GRADE evidence quality for primary outcome, sensitivity analysis, and secondary outcomes

Categaries	Outcomes	Number of studies	Risk ratio or mean difference (95 % CI)	*P*	*P* for heterogeneity	I^2^ (%)	Quality
Primary outcome Sensitivity analysis	VAP high-quality trials	4 [17, 20, 34, 42]	0.54 (0.40, 0.74)	<0.00001	0.39	0	Moderate^c^
	VAP total trials	20 [17, 20–24, 30–43]	0.55 (0.48, 0.63)	<0.00001	0.85	0	High^a^
	VAP invasive diagnosis	13 [17, 20, 22, 30, 31, 33, 35–40, 42]	0.55 (0.47, 0.65)	<0.00001	0.60	0	High^a^
	VAP excluding trials with multiple manipulations	16 [17, 22–24, 30–34, 36–43]	0.55 (0.48, 0.65)	<0.00001	0.97	0	High^a^
	VAP randomization	12 [17, 20–22, 34, 35, 37–42]	0.56 (0.47, 0.66)	<0.00001	0.37	7	High^a^
	VAP allocation concealment	5 [17, 20, 34, 39, 42]	0.56 (0.42, 0.74)	<0.00001	0.53	0	Moderate^c^
	VAP assessment blinded	8 [17, 20, 23, 31, 34, 35, 38, 42]	0.53(0.42, 0.66)	<0.00001	0.34	11	Moderate^c^
	VAP participants more than 100	10 [17, 21, 22, 30–32, 34, 38, 39, 42]	0.54(0.45, 0.65)	<0.00001	0.55	0	High^a^
	VAP intermittent SSS	9 [21–23, 30, 34, 37, 38, 42, 43]	0.52 (0.43, 0.64)	<0.00001	0.25	22	High^a^
	VAP continuous SSS	11 [20, 22, 24, 31–33, 35, 36, 39–41]	0.61(0.5, 0.73)	<0.00001	0.91	0	High^a^
Secondary outcomes	Early-onset VAP	9 [22–24, 33, 36–38, 42, 43]	0.34 (0.25, 0.47)	<0.00001	0.84	0	Moderate^c^
	Late-onset VAP	5 [22, 33, 36, 38, 42]	0.80 (0.62, 1.02)	0.07	0.17	35	Moderate^c^
	Gram-negative bacteria	6 [31, 32, 38–40, 42]	0.58(0.43, 0.77)	0.0002	0.69	0	Moderate^c^
	Gram-positive bacteria	5 [31, 33, 38, 40, 42]	0.32 (0.17, 0.61)	0.006	0.61	0	Low^b^
	ICU mortality	8 [17, 20, 30, 31, 38, 39, 41, 42]	0.98(0.85, 1.13)	0.77	0.88	0	High
	Hospital mortality	7 [17, 21, 32, 34, 37, 40, 42]	0.92 (0.80, 1.05)	0.20	0.82	0	High
	Time-to-onset of VAP^g^	7 [30–33, 37, 40, 41]	3.92 (2.56, 5.27)	<0.00001	<0.00001	92	Moderate^d^
	Duration of MV^g^	6 [20, 32, 34, 37, 38, 41]	−1.17 (–2.28, –0.06)	0.006	0.06	54	Moderate^d^
	ICU length of stay^g^	4 [32, 34, 38, 41] 37	−1.64 (–3.95, 0.66)	0.16	0.001	81	Moderate^d^
	Hospital length of stay	3 [32, 34]	−1.44 (–3.93, 1.04)	0.25	0.99	0	High
	Reintubation	4 [20, 32, 39, 42]	0.77 (0.45, 1.32)	0.34	0.19	38	Low^b^
	Tracheotomy^g^	3 [32, 38, 42]	1.14 (0.75, 1.72)	0.55	0.79	54	Low^b^

*CI* confidence interval, *GRADE* Grading of Recommendations Assessment, Development, and Evaluation, *ICU* intensive care unit, *MV* mechanical ventilation, *NA* not available, *SSS* subglottic secretion suctioning, *VAP* ventilator-associated pneumonia

^a^Total number of events is more than 300

^b^Total number of events is less than 100

^c^Total number of events is less than 300

^d^I^2^ > 50 %

^e^The total number of patients is relatively small (<500)

^f^The total number of patients is very small (100)

^g^Random-effect model

**Fig. 4:**
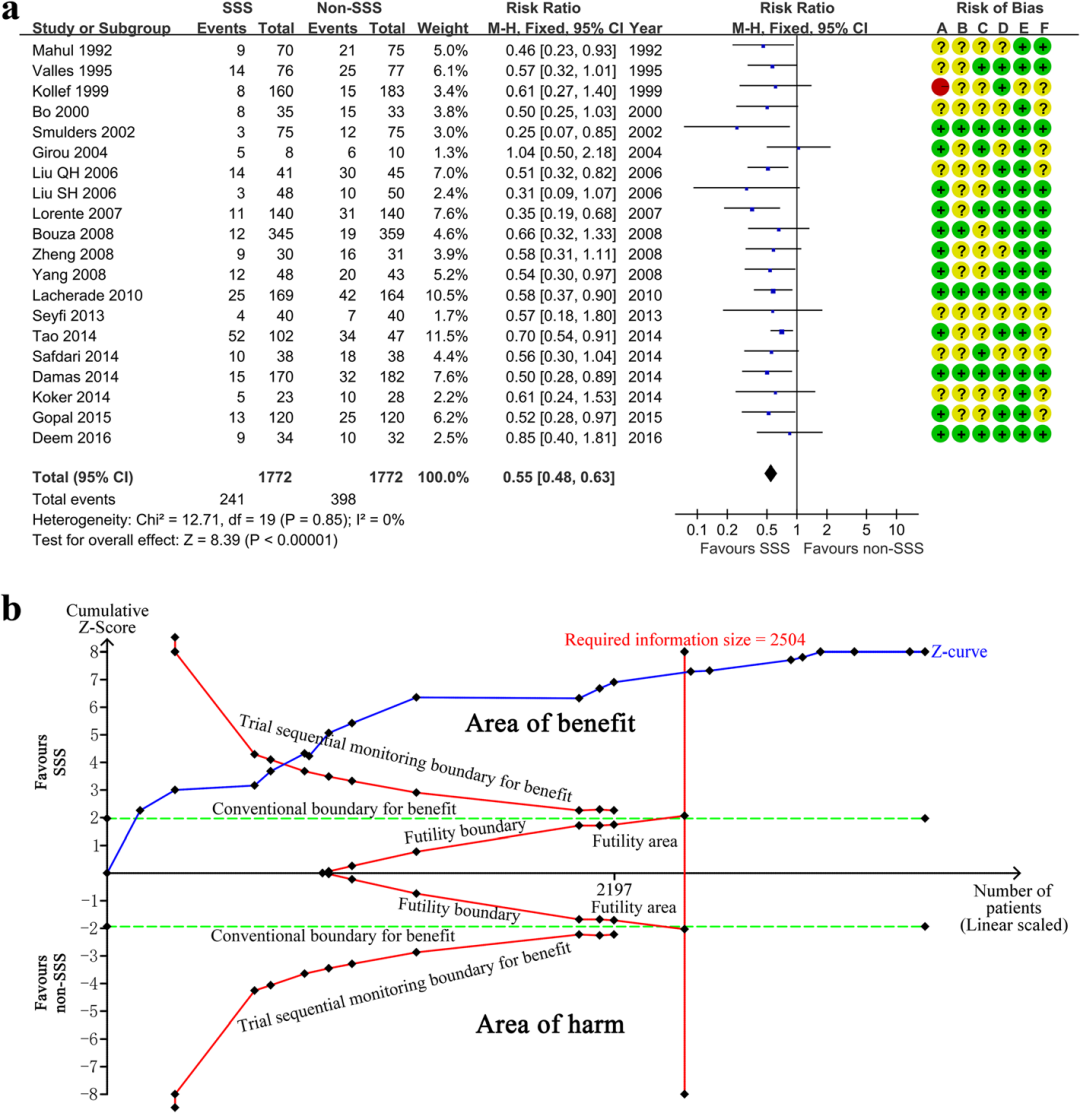
Effect of subglottic secretion suction on preventing ventilator-associated pneumonia in all included trials. **a** Forest plot comparing subglottic secretion suction (*SSS*) and non-SSS on incidence of ventilator-associated pneumonia. **b** Trial sequential analysis for incidence of ventilator-associated pneumonia with control arm event proportion of 22.5 %, relative risk reduction of 20 %, α of 5 % (two sided), β of 20 %, and *I* ^2^ = 0 %. The required information size was calculated as 2504. Z curve has across-trial sequential monitoring boundary for benefit and required information size. Risk of bias: *A* random sequence generation (selection bias), *B* allocation concealment (selection bias), *C* blinding of outcome assessment (detection bias), *D* incomplete outcome data (attrition bias), *E* selective reporting (reporting bias), *F* other bias; *CI* confidence interval, *M-H* Mantel-Haenszel

Twenty studies totalling 3544 patients provided data on incidence of VAP despite a risk of bias. Overall, subglottic secretion suction significantly prevented the incidence of VAP (RR = 0.55, 95 % CI 0.48–0.63; *p* < 0.00001; *p* for heterogeneity = 0.85, *I* ^2^ = 0 %) (Fig. 1). TSA showed that the cumulative Z curves crossed both the conventional and the trial sequential monitoring boundaries and reached the significant area, which led us to draw a conclusion. Thus, it is unlikely that further trials will change the conclusion and are not necessary (Fig. 4).

**Fig. 5:**
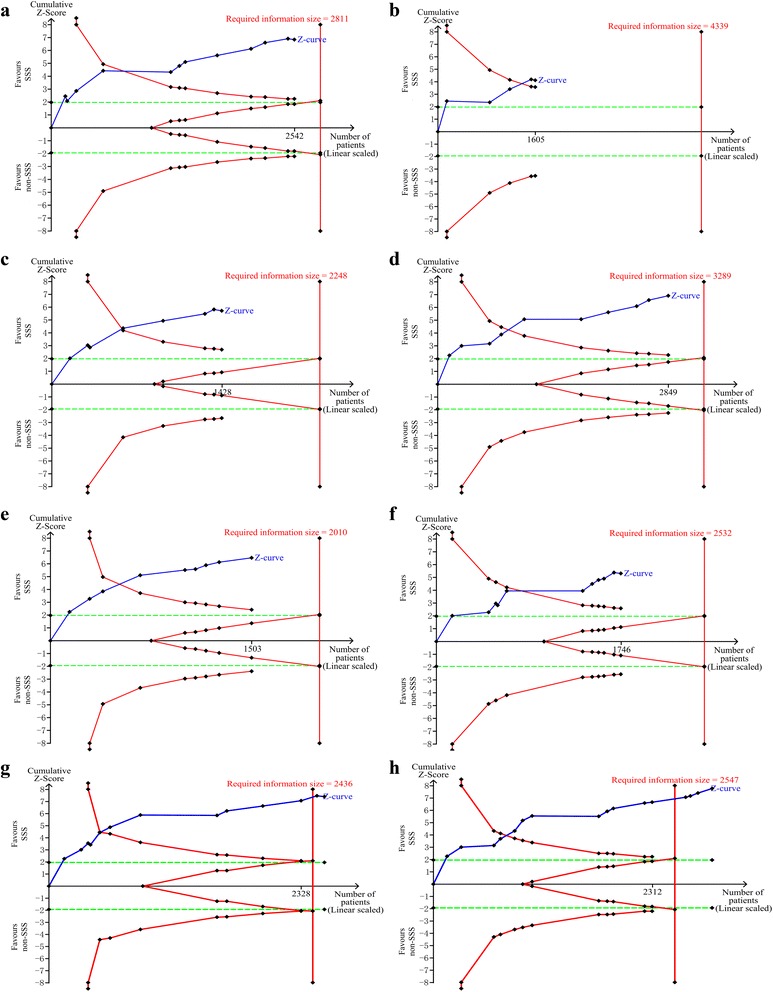
Trial sequential analysis for sensitivity analysis of incidence of ventilator-associated pneumonia. In (**a**–**h**) TSA plots, all Z curves have crossed the trial sequential monitoring boundary for benefit. **a** Trial sequential analysis in 12 appropriate randomized trials with control arm event proportion of 20.5 %, relative risk reduction of 20 %, α of 5 % (two sided), β of 20 %, and *I* ^2^ = 7 %. The required information size was calculated as 2811. **b** Trial sequential analysis in five appropriate allocation concealment trials with control arm event proportion of 14.2 %, relative risk reduction of 20 %, α of 5 % (two sided), β of 20 %, and *I* ^2^ = 0 %. The required information size was calculated as 4339. **c** Trial sequential analysis in eight appropriate assessment blinded trials with control arm event proportion of 24.5 %, relative risk reduction of 20 %, α of 5 % (two sided), β of 20 %, and *I* ^2^ = 11 %. The required information size was calculated as 2248. **d** Trial sequential analysis in 10 trials including over 100 participants with control arm event proportion of 18.0 %, relative risk reduction of 20 %, α of 5 % (two sided), β of 20 %, and *I* ^2^ = 0 %. The required information size was calculated as 3289. **e** Trial sequential analysis in nine intermittent suction trials with control arm event proportion of 26.7 %, relative risk reduction of 20 %, α of 5 % (two sided), β of 20 %, and *I* ^2^ = 22 %. The required information size was calculated as 2010. **f** Trial sequential analysis in 11 continuous suction trials with control arm event proportion of 22.3 %, relative risk reduction of 20 %, α of 5 % (two sided), β of 20 %, and *I* ^2^ = 22 %. The required information size was calculated as 2532. **g** Trial sequential analysis in 13 trials with control arm event proportion of 23.0 %, relative risk reduction of 20 %, α of 5 % (two sided), β of 20 %, and *I* ^2^ = 0 %. The required information size was calculated as 2436. **h** Trial sequential analysis in 16 trials excluding multiple manipulations studies with control arm event proportion of 16.1 %, relative risk reduction of 20 %, α of 5 % (two sided), β of 20 %, and *I* ^2^ = 0 %. The required information size was calculated as 2547. *SSS*, subglottic secretion suctioning

Sensitivity analyses were performed to evaluate only including trials using invasive diagnosis of VAP, the influence of the intervention types, risk of bias, and sample size on the combined estimates (Table 2). Some confounding interventions, such as polyurethane cuff [20, 38], continuous control of cuff pressure [21], and semi-recumbent position [35] existed in some included trials. The sensitivity analysis excluded these trials, which used other confounding interventions [20, 21, 35, 38]. The result of sensitivity analysis was stable (Table 2). Additionally, TSA of sensitivity analyses were conducted to confirm the significant results (Fig. 5).

### Secondary outcomes

SSS was significantly associated with a reduction in the incidence of early-onset VAP, gram-positive or gram-negative bacteria causing VAP, and duration of mechanical ventilation. SSS also delayed the time-to-onset of VAP. However, no significant difference was detected between SSS and non-SSS in terms of late-onset VAP, ICU mortality, hospital mortality, ICU length of stay, incidence of tracheotomy, or reintubation (Table 2). Sensitivity analyses using an invasive diagnosis of VAP in all secondary outcomes did not show additional significant results (Additional file 1: Table S1).

### GRADE

The GRADE level of evidence is high for VAP incidence, hospital mortality, and hospital length of stay, but moderate for early-onset VAP, gram-negative bacteria causing VAP, ICU mortality, time to achieve VAP, and duration of mechanical ventilation and ICU stay. Table 2 shows the GRADE evidence profiles. The main reasons for a deceasing level were inconsistency and limited sample size.

### Publication bias

For the meta-analysis of VAP including all trials no significant publication bias was observed by inspection of the funnel plot (Additional file 2: Figure S1). The Begg test and Egger test showed that no significant publication bias was detected (*p* = 0.770 and *p* = 0.051, respectively).

## Discussion

Our systematic review and meta-analysis found that SSS significantly reduced the incidence of VAP both in high-quality trials and in all included trials. The beneficial evidence was consistent in all sensitivity analysis and was validated by TSA. Meanwhile, SSS further reduced early-onset VAP, incidence of bacteria detection in airway secretion, duration of MV, and delayed time-to-onset of VAP. However, SSS did not shorten length of ICU stay, or improve ICU or hospital mortality regardless of the clinical diagnosis or invasive diagnosis of VAP.

Several previous meta-analyses have reported on the same topic, as presented in Additional file 3 (Table S2) [11–16]. Differences between the present meta-analysis and the previous ones are as follows. First, our meta-analysis included three additional trials that were published recently, and these were not included in previous meta-analyses. As the latest and most comprehensively updated meta-analysis, the present study further reinforces the earlier results of previous meta-analyses. Second, we registered the protocol of this study on PROSPERO. A registered protocol may increase the transparency and quality of meta-analysis. Third, TSA was further applied to estimate the effect more conservatively. We also used TSA of subgroup analysis and sensitivity analysis to confirm the conclusion. Fourth, the present study adds outcome data to the current body of evidence as follows: SSS may prevent early-onset VAP, reduce incidence of bacteria detection, prolong time-to-onset of VAP, and reduce duration of MV. Last, but not least, we give the evidence body level using the GRADE approach. Thus, the conclusions of this study can be clinically used and transferred to guidelines.

The present meta-analysis showed that SSS may significantly reduce the incidence of VAP. We calculated the ARR (0.0953) and NNT (10.49). This value of ARR (0.0953) meant that SSS can reduce 9.53 % of the absolute rate of VAP. NNT (10.49) means that for every 11 patients with SSS, one VAP was prevented. Additionally, we tried to answer a second question whether or not future research is needed to confirm this conclusion. Two current effective approaches, namely TSA and GRADE, were used.

Sensitivity analyses found that both intermittent and continuous suction can prevent VAP, with no significant difference between subgroups. It is difficult to determine which approach is appropriate because the current evidence is limited. Berra et al. reported that continuous suction was associated with widespread injury to the apptracheal mucosa/submucosa [44]. Thus, an intermittent approach may be beneficial for efficacy and safety. However, tracheal damage with subglottic secretion drainage is observed in animal studies but not in human data. Additionally, no side effects, such as tracheal damage, have been reported despite the large number of patients with SSS worldwide. Moreover, the trials [11, 13–15, 44] on SSS-related complications are rare, and further trials comparing intermittent and continuous suction are needed.

The present study suggested that SSS may significantly prevent early-onset VAP, but not late-onset VAP. The attributable mortality of late-onset VAP is higher than that of early-onset VAP, which weakens the impact of SSS on mortality from VAP. Dezfulian et al. suggested that SSS could prevent early-onset VAP in their meta-analysis, but late-onset VAP was not addressed in their study [11]. A meta-analysis by Wang et al. reported that SSS did not significantly reduce incidence of late-onset VAP based on three trials (591 patients) [15]. In the present meta-analysis, we included five trials (963 patients), and no significant difference was suggested. Based on the results, SSS may be suitable for patients with high risk of early-onset VAP.

Our meta-analysis also suggested that SSS can reduce incidence of bacteria detection, including both gram-negative bacteria and gram-positive bacteria causing VAP. This finding, which was not analysed in previous meta-analyses, supports the pathogenesis of VAP that leakage of fluid with bacteria passes the tracheal tube cuff toward the lungs [45–48]. Our meta-analysis also found that SSS significantly delayed the time-to-onset of VAP. According to this finding, SSS may greatly reduce incidence of VAP in patients who may have undergone early tracheal extubation. Our meta-analysis suggested that SSS can reduce the duration of MV. Interaction between the duration of MV and VAP may exist. Observational studies showed that VAP, especially early-onset VAP [49], was associated with an increased duration of MV [50]. However, most RCTs did not show a significant reduction of MV duration, whereas VAP was effectively prevented. Sample size was amplified through meta-analysis. Thus, data synthesis may introduce a different result.

This study failed to show that SSS could significantly reduce ICU and hospital mortality, as well as the length of ICU stay. There may be several reasons for this. Firstly, these outcomes may have many influencing factors or confounding bias [40, 45–48]. Secondly, VAP was associated with a 20–30 % higher risk increase of mortality [2, 42] than for non-VAP patients; thus, an ARR (0.0953) of VAP in this study may reduce the ICU mortality by 1.9–2.9 %. As the mortality of the control group was 25.6 % (as in our study), a very large sample size (at least 1940–4519 in each arm) would be needed to reach a significant result for ICU mortality [42]. Thirdly, the large database epidemiologic study of Rello et al. [3] also suggested that VAP-preventing strategies may not improve the survival rate, but they may provide other important advantages to patients, their families, and hospital systems.

SSS also has some disadvantages. The first disadvantage is the narrower inner lumen of the endotracheal tubes which may increase the airway resistance. Secondly, endotracheal tubes with SSS would result in unjustified incremental costs [51]. This limits the use of SSS. A prospective observational study showed that only 41.5 % of all intubated patients used SSS [52].

A major strength of this study is the application of Cochrane methodology and complying with the PRISMA guidelines. Moreover, our study protocol was registered in PROSPERO. To increase the robustness of this meta-analysis, TSA was conducted to evaluate the risk of random error and repetitive testing. Sensitivity analyses based on various selection criteria all obtained a significant result both in traditional meta-analysis and in TSA, which robustly supported our primary finding. Moreover, the GRADE approach was performed to give the level of evidence.

Our meta-analysis also has limitations. Firstly, the included RCTs in this meta-analysis were performed in different patient groups and various clinical settings. Therefore, the risk of introducing potential heterogeneity is present, although the detected heterogeneity is not significant. Secondly, because SSS is an obvious clinical manipulation, it could not be blinded for physicians and nurses; this may lead to unavoidable performance bias. Thirdly, confounding interventions, such as polyurethane, continuous control of cuff pressure, and semi-recumbent position, existed in some included trials. Therefore, we performed a sensitivity analysis excluding these trials which used other confounding interventions. Fortunately, the result of sensitivity analysis was stable. Fourthly, data on cost-effectiveness of SSS was unavailable in our meta-analysis. The main problem with SSS is the fact that, in some countries, a large number of patients are intubated before ICU admission with a tracheal tube without an SSS channel. Although previous retrospective studies suggested that SSS was cost-effective [53, 54], no RCT has conducted cost-effectiveness analysis. Thus, the widespread use of SSS is limited, and RCTs with cost-effectiveness analysis may be needed.

## Conclusions

This meta-analysis suggests that SSS significantly reduced the incidence of VAP, early-onset VAP, gram-positive or gram-negative bacteria causing VAP, and duration of mechanical ventilation. SSS delayed the time-to-onset of VAP. However, SSS did not show a significant difference in terms of late-onset VAP, ICU mortality, hospital mortality, ICU length of stay, incidence of tracheotomy, or reintubation. In summary, SSS is recommended to prevent VAP and to reduce ventilation length, especially in the population at high risk of early-onset VAP.

## Additional files

Additional file 1: Table S1. The secondary outcomes with VAP invasive diagnosis. (DOC 56 kb)
Additional file 2: Figure S1. Funnel plot to evaluate potential publication bias for incidence of ventilator-associated pneumonia including all trials. *RR* relative risk, *SE* standard error. (TIF 790 kb)
Additional file 3: Table S2. Comparison with previous meta-analyses. (DOCX 16 kb)

## Abbreviations

ARR: Absolute risk reduction
CI: Confidence interval
GRADE: Grading of Recommendations Assessment, Development, and Evaluation
ICU: Intensive care unit
MD: Mean difference
MV: Mechanical ventilation
NNT: Number needed to treat
PRISMA: Preferred Reporting Items for Systematic Reviews and Meta-Analyses
RCT: Randomized controlled trial
RR: Relative risk
SSS: Subglottic secretion suctioning
TSA: Trial sequential analysis
VAP: Ventilator-associated pneumonia
