# Supplementary material for: Subglottic secretion suction for preventing ventilator-associated pneumonia: an updated meta-analysis and trial sequential analysis
Source: Crit Care. 2016 Oct 28;20:353. doi: 10.1186/s13054-016-1527-7 (PMC5084404; doi:10.1186/s13054-016-1527-7)
Supplement: Additional file 1: Table S1. — The secondary outcomes with VAP invasive diagnosis. (DOC 56 kb) [file 13054_2016_1527_MOESM1_ESM.doc]

Table S1 The secondary outcomes with VAP invasive diagnosis.

| **Outcomes** | **Number of studies** | **Risk Ratio or Mean Difference [95% CI]** | **P** | **P for heterogeneity** | **I^2^(%)** |
| --- | --- | --- | --- | --- | --- |
| Early onset VAP | 6[22,33,36,37,38,42] | 0.29(0.20, 0.44) | <0.00001 | 0.98 | 0 |
| Late onset VAP | 5[22,33,36,38,42] | 0.80 (0.62, 1.02) | 0.07 | 0.17 | 35 |
| Gram-negative bacteria | 6[31,32,38,39,40,42] | 0.58(0.43, 0.77) | 0.0002 | 0.69 | 0 |
| Gram-positive bacteria | 5[31,33,38,40,42] | 0.32 (0.17, 0.61) | 0.006 | 0.61 | 0 |
| ICU mortality | 7[17,20,30,31,38,39,42] | 0.99(0.86, 1.14) | 0.92 | 0.91 | 0 |
| Hospital mortality | 5[17,21, 37,40,42] | 0.91 (0.79, 1.04) | 0.15 | 0.64 | 0 |
| Time to get VAP* | 5[30,31,33,37,40] | 5.20 (3.05, 7.34) | <0.00001 | <0.00001 | 85 |
| Duration of MV | 3[20,37,38] | -0.05 (-2.56, -2.46) | 0.97 | 0.89 | 0 |
| ICU length of stays | 1[38] | -1.40 (-5.84, 3.04) | 0.54 | NA | NA |
| Hospital length of stay | 1[37] | -2.00 (-12.23, 8.23) | 0.70 | NA | NA |
| Reintubation* | 3[20, 39,42] | 0.70 (0.19, 2.53) | 0.59 | 0.09 | 58 |
| Tracheotomy | 2[38,42] | 1.13 (0.73, 1.77) | 0.58 | 0.50 | 0 |

a Total number of events is more than 300.

b Total number of events is less than 100.

c Total number of events is less than 300.

d I^2^>50%

e The total number of patients is relatively small（<500）.

f The total number of patients is very small（100）.

*. random effect model

NA: not available; MV, mechanical ventilation.
