# Supplementary figures and images for: Subglottic secretion suction for preventing ventilator-associated pneumonia: an updated meta-analysis and trial sequential analysis
Source: Crit Care. 2016 Oct 28;20:353. doi: 10.1186/s13054-016-1527-7 (PMC5084404; doi:10.1186/s13054-016-1527-7)

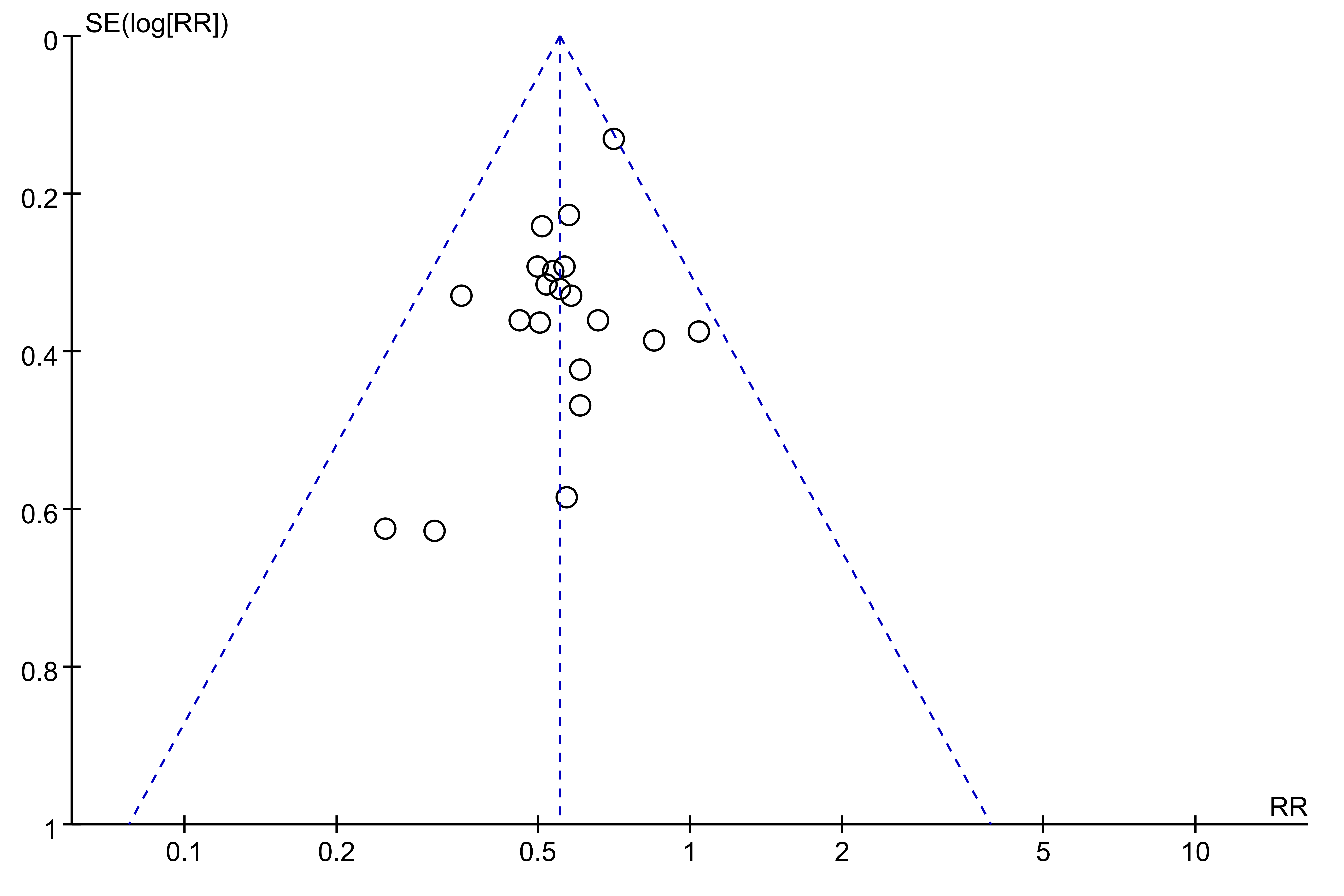

Supplement: Additional file 2: Figure S1. — Funnel plot to evaluate potential publication bias for incidence of ventilator-associated pneumonia including all trials. RR relative risk, SE standard error. (TIF 790 kb) [file 13054_2016_1527_MOESM2_ESM.tif]
