# Supplementary material for: Subglottic secretion suction for preventing ventilator-associated pneumonia: an updated meta-analysis and trial sequential analysis
Source: Crit Care. 2016 Oct 28;20:353. doi: 10.1186/s13054-016-1527-7 (PMC5084404; doi:10.1186/s13054-016-1527-7)
Supplement: Additional file 3: Table S2. — Comparison with previous meta-analyses. (DOCX 16 kb) [file 13054_2016_1527_MOESM3_ESM.docx]

| Author | Defulian/2005[11] | Muscedere/2011[14] | Leasure/2012[13] | Wang/2012[15] | Frost/2013[12] | Caroff/2016[16] | The present  meta-analysis |
| --- | --- | --- | --- | --- | --- | --- | --- |
| Number of RCTs | 5 | 13 | 13 | 10 | 9 | 17 | 20 |
| Number of participants | 896 | 2442 | 1709 | 2213 | 2839 | 3369 | 3544 |
| Search strategy until(year) | 2003 | 2011 | 2011 | 2010 | 2011 | 2015 | 2016 |
| Protocol registered | NA | NA | NA | NA | NA | NA | Applied |
| Trial sequential analysis | NA | NA | NA | NA | NA | NA | Applied |
| GRADE | NA | NA | NA | NA | NA | NA | Applied |
| Incidence of VAP (RR, 95% CI) | 0.51(0.37, 0.71) | 0.55(0.46, -0.66) | 0.52(0.43, 0.64) | 0.56(0.45, 0.69) | 0.52(0.42,0.65) | 0.58(0.51, 0.67) | 0.55 (0.48, 0.63) |
| Early onset VAP (RR, 95% CI) | 0.38(0.16, 0.88) | NA | NA | 0.23(0.13, 0.43) | NA | NA | 0.34 (0.25, 0.47) |
| Late onset VAP (RR, 95% CI) | NA | NA | NA | 1.15(0.51, 2.60) | NA | NA | 0.82 (0.59, 1.13) |
| Gram-negative bacteria (RR, 95% CI) | NA | NA | NA | NA | NA | NA | 0.61(0.45, 0.84) |
| Gram-positive bacteria (RR, 95% CI) | NA | NA | NA | NA | NA | NA | 0.36 (0.18, 0.75) |
| ICU mortality (RR, 95% CI) | NA | 1.01(0.85, 1.20) | NA | NA | 1.05(0.86, 1.28) | 0.94(0.82, 1.08) | 0.98(0.85, 1.13) |
| Hospital mortality (RR, 95% CI) | 1.1(0.8, 1.4) | 0.97(0.83, 1.13) | NA | 0.93(0.77, 1.14) | 0.96(0.81, 1.12) | 0.92(0.76,1.10) | 0.92 (0.80, 1.05) |
| Time to onset VAP(MD, 95% CI) | 6.8(5.5, 8.1) | 2.66(1.06-4.26) | 4.4(2.60, 5.47) | 3.90(2.56, 5.24) | 2.88(0.09, 5.69) | NA | 3.92 (2.56, 5.27) |
| Ventilation length(day) (MD, 95% CI) | -1.8(-1.5, -2.1) | -1.08(-2.04, -0.12) | -1.47(-2.27, -0.67) | -1.55(-2.40, -0.71) | -1.04(-2.79, 0.71) | -0.16(-0.64, 0.33) | -1.17 (-2.28, -0.06) |
| ICU length of stay(day) (MD, 95% CI) | -3(-2.1, -3.9) | -1.52(-2.94, -0.11) | -1.97(-3.91, -0.02) | -2.04(-4.18, 0.09) | NA | 0.17(-0.62, 0.95) | -1.64 (-3.95, 0.66) |

Table S2. Comparison with other previous meta-analyses

Abbreviations: CI, confidence interval; GRADE, Grading of Recommendations Assessment, Development, and Evaluation; MD, mean difference; NA, not applied; RCT, randomized controlled trial; RR, relative risk; VAP, ventilator-associated pneumonia.
